# Screening and characterization of prophages in *Desulfovibrio* genomes

**DOI:** 10.1038/s41598-018-27423-z

**Published:** 2018-06-18

**Authors:** Josicelli Souza Crispim, Roberto Sousa Dias, Pedro Marcus Pereira Vidigal, Maíra Paula de Sousa, Cynthia Canêdo da Silva, Mateus Ferreira Santana, Sérgio Oliveira de Paula

**Affiliations:** 1 Departamento de Microbiologia, Universidade Federal de Viçosa, Viçosa, 36570-900 Brazil; 2 Núcleo de Biomoléculas, Universidade Federal de Viçosa, Viçosa, Brazil; 3 Centro de Pesquisas e Desenvolvimento Leopoldo Américo Miguez de Mello, CENPES, Rio de Janeiro, Brazil; 4 Departamento de Biologia Geral, Universidade Federal de Viçosa, Viçosa, Brazil

## Abstract

Bacteria of the genus *Desulfovibrio* belong to the group of Sulphate Reducing Bacteria (SRB). SRB generate significant liabilities in the petroleum industry, mainly due to their ability to microbiologically induce corrosion, biofilm formation and H_2_S production. Bacteriophages are an alternative control method for SRB, whose information for this group of bacteria however, is scarce. The present study developed a workflow for the identification of complete prophages in *Desulfovibrio*. Poly-lysogenesis was shown to be common in *Desulfovibrio*. In the 47 genomes analyzed 53 complete prophages were identified. These were classified within the order *Caudovirales*, with 69.82% belonging to the *Myoviridade* family. More than half the prophages identified have genes coding for lysozyme or holin. Four of the analyzed bacterial genomes present prophages with identity above 50% in the same strain, whose comparative analysis demonstrated the existence of colinearity between the sequences. Of the 17 closed bacterial genomes analyzed, 6 have the CRISPR-Cas system classified as inactive. The identification of bacterial poly-lysogeny, the proximity between the complete prophages and the possible inactivity of the CRISPR-Cas in closed bacterial genomes analyzed allowed the choice of poly-lysogenic strains with prophages belonging to the *Myoviridae* family for the isolation of prophages and testing of related strains for subsequent studies.

## Introduction

Viruses have a diversity of hosts and are considered obligate intracellular parasites. Viruses that infect bacteria are referred to as bacteriophages^1^. These viruses have great abundance in the biosphere^2^, with the marine floor being the richest in bacteriophages^3^. When in contact with their host, bacteriophages release their genetic material into the cell, where they follow the lytic or lysogenic cycle^4^. The lytic cycle is characterized by viral replication and subsequent viral particle release with host cell lysis. On the other hand, the integration of the viral genome into the host genome, named the prophage, occurs in the lysogenic cycle. Prophages remain in the lysogenic state until the lytic cycle is activated through chemical and physical stresses or by spontaneous induction^5,6^. While they are present in the bacterial genome, these prophages are directly related to the genome diversity of the host cell, contributing positively or not, to bacterial fitness^7,8^. In addition to the better understanding of the involvement of bacteriophages in bacterial development, the identification and isolation of prophages allows, (i) the development of forms of bacterial control based on genetic modifications^9,10^, (ii) identification of genes of interest^11^ and (iii) infection tests in related and distinct strains^12–14^.

The replication and integration of the bacteriophage genetic material into the host strain can be avoided through the CRISPR-Cas system^15^. This system is characterized by a genomic region composed by spacers alternated with small palindromic sequences and associated with Cas proteins. Spacers are sequences related to the invader genetic material, such as bacteriophages and plasmids, inserted into this region by proteins that constitute the system. The CRISPR-Cas acts as a defense system in prokaryotes because the spacers store memory sequences against the invading target, leading to breakdown after recognition by the system^16^. Due to the small number of studies on prophages and CRISPR-Cas systems present in the Sulphate Reducing Bacteria (SRB) group, this area is of great interest to understand the involvement of bacteriophages in the diversity of the group and for future development of SRB control methods.

SRB are composed of anaerobic bacteria capable of reducing sulfate during metabolism, with hydrogen sulphide (H_2_S) as the final product^17^. These bacteria are ubiquitous in anaerobic environments, but have a negative impact on the oil industry. Additionally, H_2_S gas is toxic to workers, causes metal bio-corrosion and a loss of oil quality^18–20^. According to Rabus *et al*.^17^, there are approximately 37 bacterial genera in the SRB group. *Desulfovibrio* is the second genus that encompasses the largest number of species within this bacteria group, being characterized by Gram-negative bacteria. The first SRB was isolated in 1895 in the Netherlands by W. M. Beijerinck and named *Spirillum desulfuricans*^21^. Later, this strain was reclassified as the genus *Desulfovibrio*, based on cellular vibrio morphology and the ability to reduce sulfate. *Desulfovibrio vulgaris* is among the most well-known *Desulfovibrio* species and was isolated from clay soil, near Hildenborough (Kent, UK). This location gave its name to the strain, being the first SRB species sequenced and characterized as a model strain^22^. Another model species is *Desulfovibrio gigas*, which was isolated from a water sample from a pond in France (Etang de Berre, near Marseilles) by Jean Legall in 1963 and named for its unusual size of 11 μm^23^. Some strains are related to the petroleum industry, like as *Desulfovibrio alaskensis*, which was isolated in 1991 by E. van der Vende in an oil reservoir in Alaska, and once again named after its place of discovery^24^. The species *D. vulgaris* and *D. gigas*, although not isolated from petroleum extraction or processing environments, are used as model strains in SRB control analyses for these environments^25^.

SRB genome studies are increasing, with approximately 47 *Desulfovibrio* genomes deposited in NCBI (National Center for Biotechnology Information) to date. Thus, the objective of the present study was to verify the presence of prophages and CRISPR-Cas systems in the *Desulfovibrio* genomes. Poly-lysogeny has been shown to be common in *Desulfovibrio* strains, with complete prophages having genes encoding holins and lysozyme proteins. Prophages of the *Myoviridae* family have been shown to be the most abundant and, together with the inactivity of the CRISPR-Cas system found in some bacterial strains, the use of prophages and their genes proves to be an interesting tool for SRB control. This is the first analysis of prophages present in this important SRB genus.

## Results

### Computational identification of *Desulfovibrio* prophages

**Figure 1:**
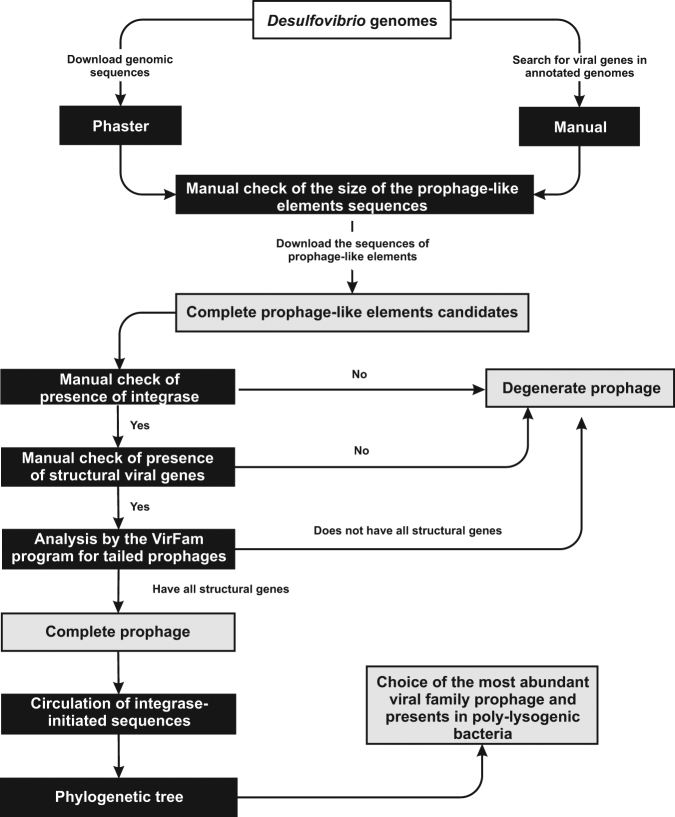
Identification and characterization strategies for *Desulfovibrio* prophages. The black rectangles represent the tools used for prophage identification and characterization. The white rectangles represent the input data and the gray rectangles represent the results obtained at each step represented by arrows.

Two prophage identification strategies were used: identification through the PHASTER program and manual search for phage related sequences in annotated genomes (Fig. 1). The PHASTER program was able to identify 109 sequences related to the prophages and the manual search led to the addition of 19 sequences, totaling 128 prophage-like elements. According to the established method, the 128 elements are present in 46 of the 47 analyzed genomes (Supplementary Table S1).

**Figure 2:**
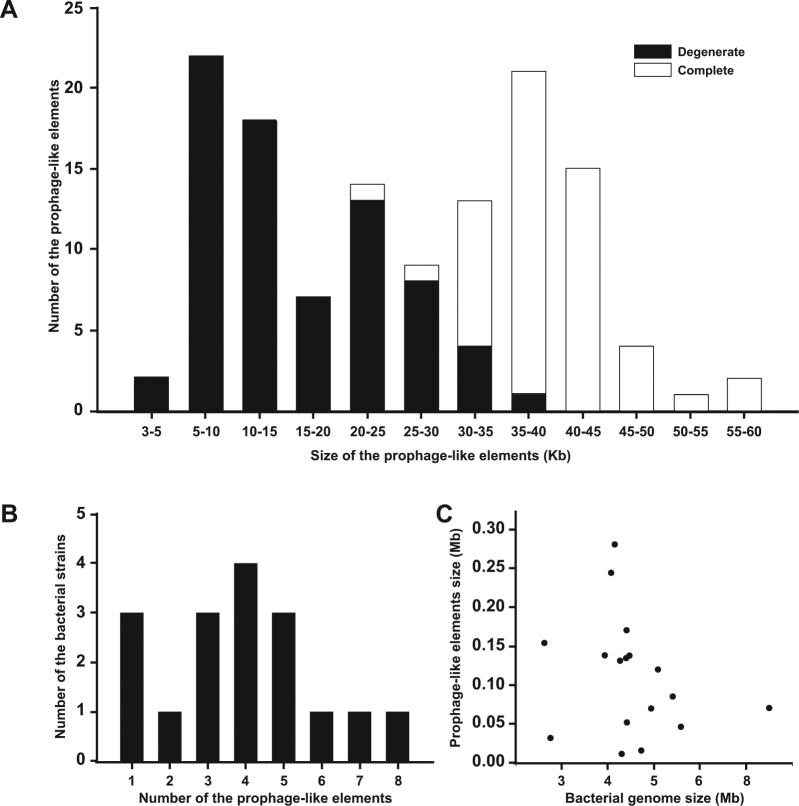
Characteristics of prophage-like elements in *Desulfovibrio*. (**A**) Distribution of prophage-like elements in 46 *Desulfovibrio* genomes in each category: degenerate and complete. The bars represent the number of elements corresponding to the size range of the sequence found. (**B**) Frequency of integration of prophage-like elements in 17 closed bacterial genomes. (**C**) Correlation between the size of closed bacterial genomes and prophage-like elements.

The identified prophage-like sequences have an average size of 25.5 kb, the smallest (3.6 kb) found in *D. vulgaris* DP4 and the largest (57.1 kb) in *D. fairfieldensis* CCUG45958 (Supplementary Table S1). 41.40% (53) of the prophage-like elements were classified as complete prophages and 58.60% (75) as degenerate prophages (Fig. 2A). Within this classification, the prophages have a variable size, with complete prophages ranging from 20 to 60 kb (mean 39.0 kb) and the degenerate prophages from 3 to 40 kb (mean 16.09 kb), with complete and degenerate prophages with the same size being observed. All the complete prophages presented structural components related to the order *Caudovirales*, which allowed the classification of 69.82% (37) of them into the *Myoviridae* family, 22.64% (12) into *Siphoviridae* and 7.54% (4) into the *Podoviridae* family (Supplementary Table S2). Of the 53 complete prophages, 38 showed genes encoding lysozyme and 29 for the holin enzyme. Both are found in 18 complete prophages (Supplementary Table S2). Complete prophages are present in 23 strains, with 4 being from human samples and 19 from environmental samples (Supplementary Table S2). Of these 23 strains, 14 are poly-lysogenic, with 5 strains presenting prophages characterized in more than one viral family (Supplementary Table S2).

From 17 strains with closed genomes (Supplementary Table S1), 81.25% (13) presented poly-lysogenicity with strains ranging from 2 to 8 prophage-like elements (Fig. 2B). The *D. vulgaris* Hildenborough strain presented the highest number of elements, with 7 of the 8 elements classified as complete, and the highest percentage of prophage-like elements in the genome (7.81%) (Supplementary Table S1). Of the four strains that did not present poly-lysogeny, the *D. piezophilus* strain CITLV30 had the lowest content of prophage-like elements (0.31%). Despite this large difference in prophage content in the closed genome strains, no correlation between bacterial genome size and prophage content was observed (R^2^ = 0.05) (Fig. 2C).

### Phylogenetic and structural relationships

**Figure 3:**
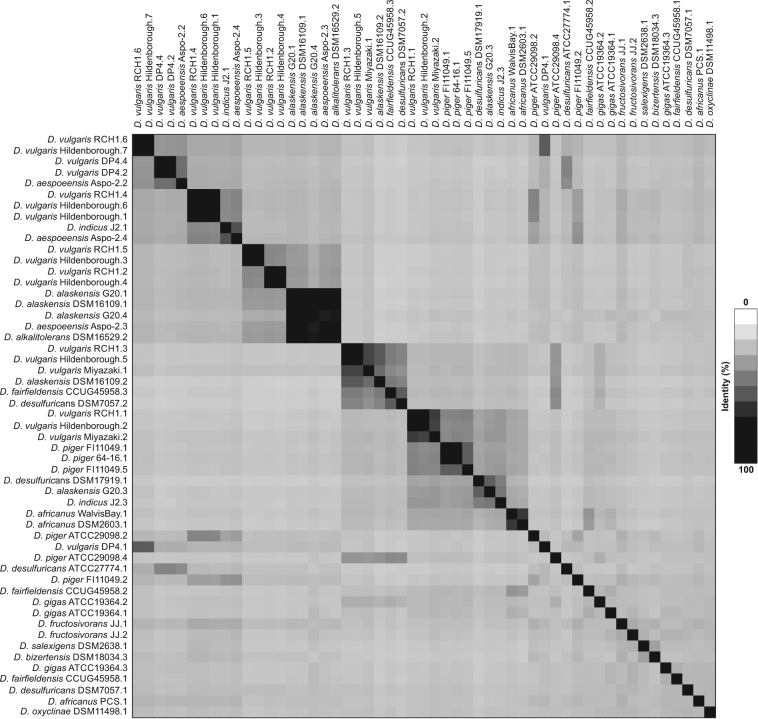
Heatmap among 53 complete *Desulfovibrio* prophages. The map describes the similarity between two pairs of sequences.

**Figure 4:**
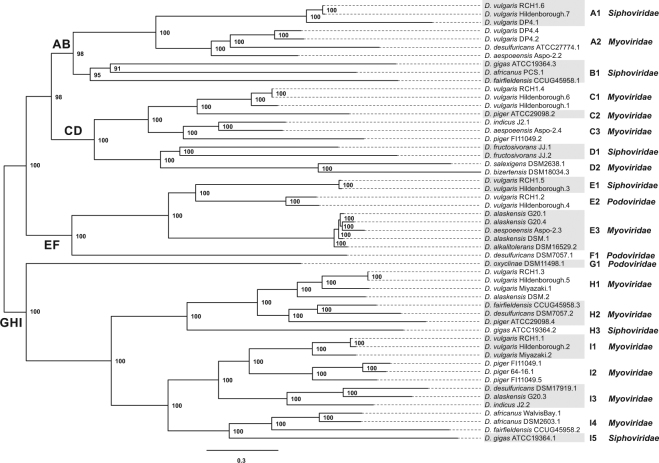
Phylogenetic relationships between complete *Desulfovibrio* prophage sequences. In the phylogenetic tree we can distinguish 9 phylogenetic groups consisting of 21 subgroups. The putative viral families of the subgroups are indicated to the right of the tree.

All 53 complete prophage sequences were aligned and a mean identity of 25.82% was found. Despite the low identity, matrix and phylogenetic tree data allowed the distinction of 9 monophyletic groups (A-I) consisting of 21 subgroups (A1-2, B1, C1-3, D1-2, E1-3, F1, G1, H1 -3 and I1-5) (Figs 3 and 4).

The phylogenetic analysis showed that complete prophages present a great diversity with prophages belonging to the same viral family forming different phylogenetic clusters, with 12 subgroups for *Myoviridae*, six for *Siphoviridae* and three for the *Podoviridae* family being found (Fig. 4). The phylogenetic tree also allowed the identification of subgroups with prophages belonging to the same bacterial species (A1, E1-2 and I1-2), which may indicate an acquisition of related prophages. In the present study, poly-lysogenesis has been shown to be common in the genus *Desulfovibrio*. Analyzing the prophages belonging to the same strain, we found that they are distributed among the phylogenetic subgroups, such as the *Desulfovibrio vulgaris* Hildenborough strain, which has elements in subgroups A1, C1, E1, E2, H1 and I1. This indicates the presence of various prophages found in a single bacterial genome.

The 9 groups were organized into 4 larger clusters (AB, CD, EF and GHI) according to the phylogenetic relationship (Fig. 4). The AB, CD, EF and GHI clusters have 479, 576, 417 and 1023 proteins distributed in 125, 161, 139 and 122 groups of orthologs, respectively (Supplementary Fig. S1). A Venn Diagram shows that only 3 groups of proteins are shared between all phylogenetic groupings, with most protein groups being specific to each cluster (Supplementary Fig. S1). The groups of orthologs shared between all clusters (AB, CD, EF and GHI) were related to transcriptional regulators (CI and Cro), structural tail protein and integrase.

**Figure 5:**
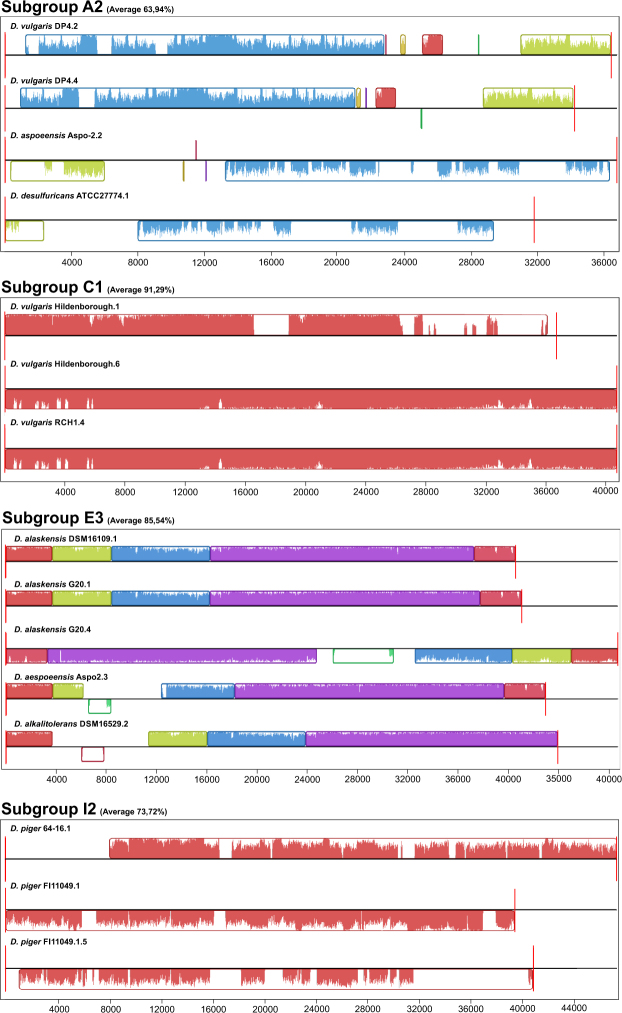
Comparison of prophage sequences of subgroups A2, C1, E3 and I2. The collinearity between the sequences is represented by the conservation of the location of the blocks in all subgroups.

Despite the diversity of prophages in poly-lysogenic strains, closely related prophages were also found in 4 strains (Fig. 4). The *D. vulgaris* DP4 strain (subgroup A2), *D. vulgaris* Hildenborough (subgroup C1), *D. alaskensis* G20 (subgroup E3) and *D. piger* FI11049 (subgroup I2) present two prophages with an identity above 50% (Fig. 3). In the D1 subgroup, prophages belonging to the same strain, *D. fructosivorans*, were also found, but these prophages have low similarity (29%) between nucleotide sequences (Fig. 3). The synteny analysis of the A2, C1, E3 and I2 subgroups showed that all sequences of complete prophages present collinearity, which is characterized by an absence of rearrangement of gene blocks (Fig. 5). The difference between prophages occurs through insertions and deletions. In the E3, the main difference between these two prophages of *D. alaskensis* G20 is in a region present in prophage 4 and absent in prophage 1. This region of prophage 4 is related to bacterial genes, demonstrating a relationship of the prophages with horizontal transference of genes.

### Analysis of CRISPR-Cas systems in *Desulfovibrio*

Seeking to understand the acquisition of prophages, the CRISPR-Cas system of *Desulfovibrio* strains with closed genomes was analyzed. Of the 17 strains analyzed, 64.7% (11) have complete CRISPR arrangements, and 1 to 5 arrangements with a total variation of 4 to 98 spacers were found (Table 1). In relation to the locus *cas*, 14 loci belonging to 5 subtypes (I-C, I-E, I-F, I-U and III-B) were found (Table 1 and Supplementary Fig. S2). These loci were found in 11 strains, with three strains presenting more than one locus, at least one of them being complete (Table 1). The *D. hydrothermalis* DSM14728 and *D. magneticus* RS-1 strains have 2 complete *cas* loci, with the subtype I-F being common to both strains, and subtype III-B and I-C present in each strain, respectively. The *D. gigas* ATCC19364 strain has two *cas* loci, the subtype I-F being classified as complete and the subtype U-I as incomplete due to its not presenting Cas8, Cas4/Cas1 and Cas2 proteins, according to the classification given by Makarova *et al*.^16^.

**Table 1:** CRISPR-Cas system in closed genomes of *Desulfovibrio*.

N°	Strain	N° of prophage-like elements	CRISPR arrays (n° of spacers)	CRISPR-Cas systems type	Putative activity
1	*D. aespoeensis* Aspo-2	4	ND	ND	Inactive
2	*D. africanus* Walvis Bay	4	ND	ND	Inactive
3	*D. alaskensis* G20	4	1 (19)	I-E	Active
4	*D. desulfuricans* ND132	3	1 (98)	I-C	Active
5	*D. desulfuricans* ATCC 27774	1	2 (29)	I-E	Active
6	*D. fairfieldensis* CCUG45958	5	Questionable	ND	Inactive
7	*D. gigas* ATCC19364	3	1 (4)	I-F e I-U Incomplete	Active
8	*D. hydrothermalis* DSM14728	3	5 (35)	III-B e I-F	Active
9	*D. indicus* J2	2	1 (19)	I-C	Active
10	*D. magneticus* RS-1	5	1 (30)	I-C e I-F	Active
11	*D. piezophilus* C1TLV30	1	Questionable	ND	Inactive
12	*D. piger* FI11049	5	Questionable	ND	Inactive
13	*D. salexigens* DSM2638	1	Questionable	ND	Inactive
14	*D. vulgaris* DP4	6	1 (44)	I-C	Active
15	*D. vulgaris* Hildenborough	8	1 (27)	I-C	Active
16	*D. vulgaris* Miyazaki	4	2 (54)	I-C	Active
17	*D. vulgaris* RCH1	7	1 (27)	I-C	Active

The type I-C *cas* locus of the DP4, Hildenborough and RCH1 strains of *D. vulgaris* have 97% identity. This result differs from the comparison with the *cas* locus of the *D. vulgaris* Miyazaki strain, which presents coverage of only 1% in relation to the loci of the other strains of the species. Unlike *D. vulgaris*, *D. desulfuricans* strains present different types of *cas* loci.

We are unable classify the activity of the system using only *in silico* analysis, however the complete CRISPR-Cas systems were classified as possibly active, being found in 64.70% of the closed genome strains (Table 1). No positive correlation was found between the number of spacers in the strains with possibly active CRISPR-Cas and the extent of the prophage content. The CRISPR-Cas system characteristics of the closed genomes were re-evaluated by the CRISPRone software (Supplementary Table S3), confirming previous findings.

## Discussion

The *in silico* workflow developed in the present study allowed the identification of 128 prophage-like elements in 46 *Desulfovibrio* genomes. However, other prophage-like elements can be identified in the future because most of the genomes used in the analysis (56.60%) were deposited in contigs form and without gene annotation, as with some strains identified in this study that did not present prophage-like elements (*D. gracilis* DSM 16080). Of the 17 closed *Desulfovibrio* genomes, 81.25% (13) present poly-lysogeny. No relationship was found between the genomic size of poly-lysogenic strains and the content of prophage-like elements that they possess. On the other hand, this data may be modified due to the low number of closed genomes available in the database. In contrast, a positive correlation was found in other bacterial species such as *Cronobacter sakazakii*, where the increase of bacterial genomes is found to contain a greater number of prophages^26,27^. The maximum value of the genomic viral content was 7.8% in *D. vulgaris* Hildenborough. A maximum value of 20% viral content has already been detected in *Borrelia burgdorferi*^28^. Some of the prophages of *D. vulgaris* Hildenborough have already been described in the literature^12,29^. Handley *et al*.^29^ described 50 nm icosahedral and contractile tail structures present in the supernatant of the strain induced by mitomycin C. Walker *et al*.^12^ used a closely related *D. vulgaris* DePue strain as a recipient for prophages induced by mitomycin C of the *D. vulgaris* Hildenborough, finding structures with an icosahedral head of 50 and 100 nm in diameter. This work is an indication that the prophage-like elements of *Desulfovibrio* are induced by mitomycin C, with its isolation from related strains being possible. Additionally, the sequence analysis of these two related strains showed that the DePue shares only one of the seven phage-related elements present in the Hildenborough, demonstrating that the genetic diversity of these strains is mainly related to the regions of the prophages^7^.

53 elements were classified as complete, characterizing possible *Desulfovibrio* prophages. The 75 elements classified as degenerate may be related to gene transfer agents^30^. The complete prophages showed an average size of 39 kb, which is close to that of caudal prophages present in the bacterial *Enterobacteriaceae* family, with approximately 40–50 Kb^31^. In addition, 71.7% of the prophages have genes encoding lysozymes and 54.7% have genes encoding holins. Lysozymes are peptidoglycan hydrolases and holins cause plasmatic membrane damage, the action of these enzymes leading to cell disruption^11,32^. The presence of these enzymes characterizes these phages as infective, highlighting an attractive feature for their isolation by induction and infection assays with different strains, as seen by Walker *et al*.^12^. Additionally, these genes can be used for the development of bacterial control tools, such as protein expression in bacterial systems for subsequent environmental application^11^.

Complete prophages found have genes related to the tail, being classified into three families in the order *Caudovirales*, with 69.82% of the prophages belonging to the *Myoviridae* family. Poly-lysogeny was found in different phage families in the same bacterial genome. The presence of prophages belonging to the different viral families is also seen in *Vibrio campbellii*, which present four complete prophages, two related to the *Myoviridae* and two to the *Inoviridae* family^33^. The classification by viral family performed in this study was according to the proteins of head and tail connectivity, a parameter used by the VirFam program. Sequence alignment was performed using the total prophage sequences. The sequences can share other genes, such as integrases, transcriptional regulators and tail proteins, as found in OrthoVenn results (Supplementary Fig. S1). The sharing of these other genes may explain the diversification of viral families in the phylogenetic tree (Fig. 4). Although most of the complete prophages have genes encoding holins and lysozymes, no shared orthologous protein was found among the orthologous protein in intersectional groups, indicating that the *Desulfovibrio* prophages exhibit significant protein diversity (Supplementary Fig. S1). This result indicates the need for several holin and lysozyme conjunctions for the control of *Desulfovibrio* strains. In addition, many hypothetical genes are also found among the complete prophages, as described for other phages^34,35^. The analysis of these genes may promote a better understanding of the prophage-host interactions and reveal other interesting genes.

Although a great diversity of complete prophages were found, the presence of similar elements within the same strain was also seen, which was analyzed by synteny analysis (Fig. 5). Related prophages were found in *D. vulgaris* DP4 (subgroup A2), *D. vulgaris* Hildenborough (subgroup C1), *D. alaskensis* G20 (subgroup E3) and *D. piger* FI111049 (subgroup I2) (Fig. 5). Although the *D. alaskensis* G20 and DSM16109 strains show prophages with 95.16% identity, the DSM16109 does not present related prophages in its genome (Fig. 5). The *D. alaskensis* DSM16109 is deposited in contig form, which may mask the presence of another similar prophage. The same is true for *D. piger* 64-16, which is deposited in contig form. Differently, the *D. vulgaris* RCH1 has a closed genome and did not present a related prophage, such as the strain of the same species *D. vulgaris* Hindenborough (Fig. 5). The results showed that some prophages found in the same species remain unchanged between the strains, while others have some changes at the nucleotide level (Fig. 5). Selective pressures maintain these prophages in the genome, therefore having some beneficial characteristics for the host^31^. The same was found for *Helicobacter pylori* strains, whose prophages showed low variation between strains from the same species^35^. On the other hand, the acquisition of related prophages is a characteristic of the strain and not of the species in *Desulfovibrio*. One factor that may be involved in this acquisition is the CRISPR-Cas system, whose spacers have a wide variety of strains, as found for *Salmonella*^36^.

*Desulfovibrio* strains mostly have type I *cas* loci, but type III was also found in one of the 17 strains analyzed with closed genomes. The presence of more than one *cas* locus allows the system to have different targets, such as type-I and type-II that have DNA strands and type III that have RNA strands as a target^37^. Three strains presented more than one *cas* locus. This allows the absence of a given gene at one locus to be compensated for by the presence of a related gene at another locus^16^, as with *D. gigas* ATCC19364, which presents one of these incomplete *cas* loci. The *cas* locus of the subtype I-C has been shown to be the most common among the *Desulfovibrio* strains, representing 50% (7) of the total *cas* loci observed (Table 1 and Supplementary Fig. S2). Although it is the most common, this subtype showed variability between strains from the same species, as found for *D. vulgaris* (Table 1). In addition, the *D. desulfuricans* species presents *cas* loci of different subtypes between the two analyzed strains (Table 1). This demonstrates that variability of *cas* loci is common within the same species.

The absence of correlation between the number of spacers in possibly active CRISPR-Cas and the size of the prophage content found in *Desulfovibrio* strains was demonstrated in other bacterial strains^26^. On the other hand, *Cronobacter sakazakii* strains presented lower prophage content with the greater presence of spacers^27^. This relationship found in *C. sakazakii* demonstrates the involvement of the CRISPR system in the protection against bacteriophages, which was not found in *Desulfovibrio* strains, according to the closed genomes analysed in this work. The spacer composition of the CRISPR system was also analyzed by Walker *et al*.^7^ in *D. vulgaris* DePue, the strain used as receptor for induced prophages of *D. vulgaris* Hildenborough. The authors found no similarity between the spacers and the prophages, a feature that allowed the infection of the recipient bacterium by prophages of the Hildenborough strain, since they were not recognized by the CRISPR-Cas system. The presence of prophages in bacteria with important role in the food industry has a negative aspect since that can be induced and follow the lytic cycle^13^. In the present study, the presence of these prophages in the strains analyzed is of great importance for the discovery of phages that can be used in SRB control.

## Conclusions

Given these results, the attempt to isolate prophages using nearby receptor host cells is feasible for this group, since part of the strains with closed genomes present the possibly inactive CRISPR-Cas system. There is also an indication that the spacers of the *Desulfovibrio* CRISPR-Cas system do not protect against bacteriophages. Additionally, the presence of related prophages in different strains demonstrated to be common, indicates that a bacteriophage could have the ability to infect strains of the same bacterial genus. All these characteristics demonstrate that bacteriophages have great potential for SRB control, because a single bacteriophage could infect different strains without being recognized by the CRISPR-Cas system. Some bacteriophages can be isolated by inducing prophages inserted in bacterial genomes, using related bacterial species as new host. The strains that show the greatest potential for prophage isolation are those that present the highest number of complete prophages belong to the *Myoviridade* family, which is the most common family in the bacterial genus. Among the strains with this potential are *D. vulgaris* (Myiazaki, Hildenborough, RCH1 and DP4), *D. piger* FI11049, *D. fairfieldenses*, *D. alaskensis* G20 and *D. aespoeensis*. Although the strains *D. piger* and *D. fairfieldenses* are of human origin, they present prophages that can be used for control of bacteria of environmental origin. In addition, many genus related to holins and lysozymes have been identified, which can be used for the development of control tools. On the other hand, the function of many genes of the prophages characterized in this study are not known, representing a vast reservoir of new information to be determined.

## Methods

### Identification of prophages and CRISPR-Cas system

Forty-seven genomes belonging to 33 species of the genus *Desulfovibrio* were downloaded from NCBI Genome database (https://www.ncbi.nlm.nih.gov/genome/), with 17 complete genome sequences. The presence of prophages in the genomes was initially verified using the PHASTER (PHAge Search Tool Enhanced Release) (http://phaster.ca/) web server^38^. The prophage sequences identified by PHASTER were manually inspected using the integrase position and the last phage-related gene as the criterion to determine the genome boundaries.

The annotation of complete bacterial genomes was also manually inspected by text-mining to screen for the presence of other phage-related regions containing proteins such as integrase, tail and capsid. Prophage sequences were screened for the presence of genes encoding integrase and structural genes, with the hypothetical proteins being further analyzed by PSI-BLAST(https://blast.ncbi.nlm.nih.gov/Blast.cgi?CMD=Web&PAGE=Proteins&PROGRAM=blastp&RUN_PSIBLAST=on)^39^. The presence of structural genes in tailed prophage sequences was verified by the VirFam (http://biodev.extra.cea.fr/virfam/) web server^40^. The prophages, which in addition to integrase, have all structural genes for classification within the families *Myoviridae*, *Siphoviridae* and *Podoviridae*, were classified as complete prophages. The prophages that do not have integrase or that show integrase also in addition to an absence of genes related to the viral structure were classified as degenerate prophages.

CRISPR-Cas systems were detected by the CRISPRFinder (http://crispr.i2bc.paris-saclay.fr/Server/) program^41^ and *cas* genes by manual analysis of closed genomes. The results obtained by these analyzes were confirmed by the CRISPRone program (http://omics.informatics.indiana.edu/CRISPRone/).

### Genomic analysis of complete prophages

The complete prophage sequences were manually analyzed using Geneious version 9.1 (Biomatters Lts), adjusting the direction of nucleotide sequences and opening the genomes for the integrase gene. Multiple sequence alignment was performed on Geneious using the ClustalW algorithm with standard parameters^42^. A pairwise identity matrix was also calculated by Geneious from the alignment of genome sequences.

The GTR + G model was selected by jModeltest version 2.1.10^43^ as the best fit nucleotide substitution model for phylogenetic analysis. The phylogenetic tree was obtained by Bayesian Inference using the MrBayers version 3.2.6^44^. A Bayesian Markov Chain Monte Carlo (MCMC) analysis was performed in two runs with 5000000 generations. The parameter convergence was analyzed and 10% of the trees generated were burnt to produce the consensus tree.

For comparative purposes, the proteins shared between the phage groups that were separated in the phylogenetic tree, were identified using OrthoVenn (http://www.bioinfogenome.net/OrthoVenn/) web server^45^. In addition, a comparative genomic analysis between phages from each group was performed using Mauve version 2.3.1^46^.

## Electronic supplementary material

Supplementary information
