# Supplementary material for: Screening and characterization of prophages in Desulfovibrio genomes
Source: Sci Rep. 2018 Jun 18;8:9273. doi: 10.1038/s41598-018-27423-z (PMC6006170; doi:10.1038/s41598-018-27423-z)
Supplement: Supplementary file 1 — Supplementary information [file 41598_2018_27423_MOESM1_ESM.docx]

**Supplementary information**

**Screening and characterization of prophages in *Desulfovibrio* genomes**

Josicelli Souza Crispim^1^, Roberto Sousa Dias^1^, Pedro Marcus Pereira Vidigal^2^, Maíra Paula de Sousa^3^, Cynthia Canêdo da Silva^1^, Mateus Ferreira Santana^1^, Sérgio Oliveira de Paula^4^*

^1^ Departamento de Microbiologia, Universidade Federal de Viçosa, Viçosa 36570-900, Brazil.

^2^ Núcleo de Biomoléculas, Universidade Federal de Viçosa, Viçosa, Brazil.

^3^ Centro de Pesquisas e Desenvolvimento Leopoldo Américo Miguez de Mello, CENPES, Rio de Janeiro, Brazil.

^4^ Departamento de Biologia Geral, Universidade Federal de Viçosa, Viçosa, Brazil.

**Supplementary Table S1** Prophage-like elements of *Desulfovibrio*

| **N°** | **Strain** | **Acess number** | **GC%** | **Prophage-like elements** | **% Genome** | | **Contig (location)** | | **Size (pb)** | **Classification** | **GC %** | **CDS** |
| --- | --- | --- | --- | --- | --- | --- | --- | --- | --- | --- | --- | --- |
| 1 | *D. vulgaris* Hildenborough | NC_002937.3 | 63.1 | 1 | 1,02 | 7,81 | | 233752-270425 | 36674 | Complete | 63.7 | 48 |
| 2 |  |  |  | 2 | 0,99 |  |  | 1205411-1241031 | 35621 | Complete | 64.8 | 47 |
| 3 |  |  |  | 3 | 1,10 |  |  | 1556492-1595902 | 39411 | Complete | 58.2 | 52 |
| 4 |  |  |  | 4 | 1,02 |  |  | 1770879-1807430 | 36552 | Complete | 59.9 | 45 |
| 5 |  |  |  | 5 | 0,98 |  |  | 2799302-2834336 | 35035 | Complete | 64.9 | 48 |
| 6 |  |  |  | 6 | 1,13 |  |  | 2936472-2977150 | 40679 | Complete | 62.6 | 54 |
| 7 |  |  |  | 7 | 1,24 |  |  | 2250828-2295194 | 44367 | Complete | 60.8 | 51 |
| 8 |  |  |  | 8 | 0,33 |  |  | 2714953-2726897 | 11945 | Degenerate | 67.6 | 17 |
| 9 | *D. vulgaris* RCH1 | NC_017310.1 | 63.1 | 1 | 1,14 | 7,18 | | 1207755-1248036 | 40282 | Complete | 63.1 | 52 |
| 10 |  |  |  | 2 | 1,05 |  |  | 1760491-1797698 | 37208 | Complete | 60.1 | 49 |
| 11 |  |  |  | 3 | 0,99 |  |  | 2802434-2837468 | 35035 | Complete | 65.9 | 49 |
| 12 |  |  |  | 4 | 1,15 |  |  | 2939604-2980282 | 40679 | Complete | 62.6 | 53 |
| 13 |  |  |  | 5 | 0,98 |  |  | 1987316-2022273 | 34958 | Complete | 59.0 | 47 |
| 14 |  |  |  | 6 | 1,51 |  |  | 2254349-2297214 | 42866 | Complete | 61.0 | 45 |
| 15 |  |  |  | 7 | 0,36 |  |  | 2718253-2731058 | 12806 | Degenerate | 67.1 | 19 |
| 16 | *D. piger*  FI11049 | NZ_LT630450.1 | 64.2 | 1 | 1.40 | 5,44 | | 25995-65412 | 39418 | Complete | 60.2 | 50 |
| 17 |  |  |  | 2 | 1,51 |  |  | 148736-191335 | 42600 | Complete | 46.8 | 56 |
| 18 |  |  |  | 3 | 0,25 |  |  | 777899-785196 | 7298 | Degenerate | 48.1 | 7 |
| 19 |  |  |  | 4 | 0,83 |  |  | 786568-810100 | 23533 | Degenerate | 63.1 | 31 |
| 20 |  |  |  | 5 | 1,45 |  |  | 430305-471180 | 40876 | Complete | 62.0 | 50 |
| 21 | *D. piger* ATCC29098 | NZ_ABXU00000000.1 | 63.0 | 1 | 1,33 | 5,16 | | 3.3:858-37806 | 37806 | Degenerate | 63.2 | 50 |
| 22 |  |  |  | 2 | 1,34 |  |  | 4.1:16868-54731 | 37864 | Complete | 62.1 | 52 |
| 23 |  |  |  | 3 | 0,22 |  |  | 4.3:1-6352 | 6352 | Degenerate | 69.3 | 8 |
| 24 |  |  |  | 4 | 1,24 |  |  | 4.4:1-35308 | 35309 | Complete | 59.2 | 51 |
| 25 |  |  |  | 5 | 1,03 |  |  | 4.5:1-29333 | 29333 | Degenerate | 58.5 | 48 |
| 26 | *D. fairfieldensis* CCUG45958 | NZ_CP014229.1 | 60.9 | 1 | 1,54 | 4,58 | | 1076345-1133484 | 57140 | Complete | 59.6 | 63 |
| 27 |  |  |  | 2 | 1,04 |  |  | 1555216-1593958 | 38473 | Complete | 62.5 | 53 |
| 28 |  |  |  | 3 | 0,82 |  |  | 2149353-2186193 | 30416 | Complete | 61.9 | 36 |
| 29 |  |  |  | 4 | 0,59 |  |  | 263020-284989 | 21970 | Degenerate | 58.3 | 18 |
| 30 |  |  |  | 5 | 0,59 |  |  | 484160-506251 | 22092 | Degenerate | 64.3 | 25 |
| 31 | *D. vulgaris* DP4 | NC_008751.1 | 63.0 | 1 | 1,23 | 3,96 | | 1272991-1315732 | 42742 | Complete | 61.6 | 50 |
| 32 |  |  |  | 2 | 1,05 |  |  | 1745393-1781803 | 36411 | Complete | 61.6 | 48 |
| 33 |  |  |  | 3 | 0,10 |  |  | 1779304-1782906 | 3603 | Degenerate | 58.1 | 7 |
| 34 |  |  |  | 4 | 0,98 |  |  | 3198595-3232809 | 34215 | Complete | 62.3 | 42 |
| 35 |  |  |  | 5 | 0,34 |  |  | 1068859-1080736 | 11878 | Degenerate | 57.7 | 14 |
| 36 |  |  |  | 6 | 0,26 |  |  | 1271645-1280709 | 9065 | Degenerate | 59.3 | 13 |
| 37 | *D.* *alaskensis* G20 | NC_007519.1 | 57.8 | 1 | 1,10 | 3,67 | | 939853-980902 | 41050 | Complete | 63.1 | 42 |
| 38 |  |  |  | 2 | 0,20 |  |  | 2787989-2795585 | 7597 | Degenerate | 47.8 | 9 |
| 39 |  |  |  | 3 | 1,07 |  |  | 3331749-3371978 | 40230 | Complete | 55.2 | 52 |
| 40 |  |  |  | 4 | 1,30 |  |  | 1918124-1966812 | 48689 | Complete | 60.7 | 50 |
| 41 | *D. gigas* ATCC19364 | NC_022444.1 | 63.8 | 1 | 1,21 | 3,62 | | 990478-1035433 | 44956 | Complete | 65.1 | 56 |
| 42 |  |  |  | 2 | 0,89 |  |  | 2474033-2507188 | 33156 | Complete | 63.1 | 42 |
| 43 |  |  |  | 3 | 1,52 |  |  | 2127703-2183892 | 56190 | Complete | 54.9 | 52 |
| 44 | *D. aespoeensis* Aspo-2 | NC_014844.1 | 62.6 | 1 | 0,36 | 3,59 | | 352121-365295 | 13175 | Degenerate | 64.9 | 8 |
| 45 |  |  |  | 2 | 1,01 |  |  | 474227-510996 | 36770 | Complete | 61.8 | 51 |
| 46 |  |  |  | 3 | 1,18 |  |  | 1775493-1818421 | 42929 | Complete | 42.9 | 44 |
| 47 |  |  |  | 4 | 1,04 |  |  | 2595084-2633147 | 38064 | Complete | 38.0 | 58 |
| 48 | *D. vulgaris* Miyazaki | NC_011769.1 | 67.1 | 1 | 0,91 | 2,94 | | 864144-901131 | 36988 | Complete | 65.2 | 43 |
| 49 |  |  |  | 2 | 0,97 |  |  | 1558929-1598331 | 39403 | Complete | 65.2 | 46 |
| 50 |  |  |  | 3 | 0,56 |  |  | 2016554-2039449 | 22896 | Degenerate | 64.4 | 30 |
| 51 |  |  |  | 4 | 0,50 |  |  | 1302496-1322867 | 20372 | Degenerate | 70.2 | 22 |
| 52 | *D. desulfuricans* DSM7057 | NZ_FPIW00000000.1 | 57.3 | 1 | 1,35 | 2,89 | | 5:2749-46467 | 43719 | Complete | 55.2 | 54 |
| 53 |  |  |  | 2 | 0,88 |  |  | 8:58080-86702 | 28623 | Complete | 58.2 | 39 |
| 54 |  |  |  | 3 | 0,39 |  |  | 50:8532-21340 | 12809 | Degenerate | 60.6 | 12 |
| 55 |  |  |  | 4 | 0,27 |  |  | 80:3-9082 | 9082 | Degenerate | 58.1 | 13 |
| 56 | *D. fructosivorans* JJ | NZ_AECZ00000000.1 | 63.9 | 1 | 0,79 | 2,59 | | 49:33508-70620 | 37113 | Complete | 62.4 | 48 |
| 57 |  |  |  | 2 | 1,08 |  |  | 49:138295-188970 | 50676 | Complete | 61.3 | 64 |
| 58 |  |  |  | 3 | 0,72 |  |  | 55:63769-97635 | 33867 | Degenerate | 64.2 | 48 |
| 59 | *D. alkalitolerans* DSM16529 | NZ_ATHI00000000.1 | 64.5 | 1 | 1,11 | 2,48 | | 12:30050-65856 | 35807 | Degenerate | 62.6 | 47 |
| 60 |  |  |  | 2 | 1,37 |  |  | 02:19980-63856 | 43877 | Complete | 62.1 | 45 |
| 61 | [*D. desulfuricans* DSM 17919](https://www.ncbi.nlm.nih.gov/genome/15717?genome_assembly_id=295293) | NZ_FQZR00000000.1 | 45.1 | 1 | 0,88 | 2,27 | | 2:441837-473030 | 31194 | Complete | 49.5 | 47 |
| 62 |  |  |  | 2 | 0,60 |  |  | 2:1139634-1160999 | 21366 | Degenerate | 47.1 | 29 |
| 63 |  |  |  | 3 | 0,79 |  |  | 11:4474-32796 | 28323 | Degenerate | 48.0 | 37 |
| 64 | *D. africanus* [DSM2603](https://www.ncbi.nlm.nih.gov/genome/3152?genome_assembly_id=172938) | NZ_AULZ00000000.1 | 61.1 | 1 | 0,74 | 2,14 | | 5:86038-118974 | 32937 | Complete | 65.4 | 47 |
| 65 |  |  |  | 2 | 0,59 |  |  | 7:81990-108084 | 26095 | Degenerate | 64.0 | 33 |
| 66 |  |  |  | 3 | 0,54 |  |  | 10:45240-69368 | 24129 | Degenerate | 63.2 | 29 |
| 67 |  |  |  | 4 | 0,27 |  |  | 12:47622-59836 | 12215 | Degenerate | 65.4 | 19 |
| 68 | [*D. africanus* Walvis Bay](https://www.ncbi.nlm.nih.gov/genome/3152?genome_assembly_id=172937) | NC_016629.1 | 61.4 | 1 | 0,84 | 2,00 | | 727103-762542 | 35440 | Complete | 64.2 | 52 |
| 69 |  |  |  | 2 | 0,63 |  |  | 2133499-2160143 | 26645 | Degenerate | 65.0 | 32 |
| 70 |  |  |  | 3 | 0,29 |  |  | 3893257-3905761 | 12505 | Degenerate | 61.5 | 10 |
| 71 |  |  |  | 4 | 0,24 |  |  | 2790428-2800848 | 10421 | Degenerate | 55.7 | 10 |
| 72 | *D. bizertensis* DSM 18034 | NZ_FUYA00000000.1 | 52.2 | 1 | 0,45 | 1,95 | | 1:283510-298460 | 14951 | Degenerate | 55.9 | 21 |
| 73 |  |  |  | 2 | 0,40 |  |  | 4:1-13153 | 13153 | Degenerate | 55.9 | 19 |
| 74 |  |  |  | 3 | 1,10 |  |  | 11:60957-97045 | 36089 | Complete | 49.9 | 36 |
| 75 | *D. piger* 64-16 | MNTW00000000.1 | 63.7 | 1 | 1,84 | 1,84 | | 1:22449-69767 | 47319 | Complete | 58.3 | 57 |
| 76 | *D. alaskensis* DSM16109 | NZ_AXWQ00000000.1 | 58.0 | 1 | 1,14 | 1,82 | | 1:145720-186292 | 40573 | Complete | 63.2 | 42 |
| 77 |  |  |  | 2 | 0,68 |  |  | 6:317764-342216 | 24453 | Complete | 58.5 | 30 |
| 78 | *D. indicus* J2 | NZ_CP014206.1 | 63.5 | 1 | 0,92 | 1,74 | | 19149-56014 | 36866 | Complete | 64.7 | 55 |
| 79 |  |  |  | 2 | 0,82 |  |  | 1479948-1512740 | 32793 | Complete | 61.9 | 50 |
| 80 | *D. bastinii* DSM16055 | NZ_AUCX00000000.1 | 43.1 | 1 | 0,31 | 1,62 | | 8:228603-240880 | 12278 | Degenerate | 45.9 | 19 |
| 81 |  |  |  | 2 | 0,79 |  |  | 22:50-31249 | 31200 | Degenerate | 45.0 | 17 |
| 82 |  |  |  | 3 | 0,52 |  |  | 31:734-21301 | 20568 | Degenerate | 41.4 | 26 |
| 83 | *D. litoralis* DSM11393 | NZ_FRDI00000000.1 | 36.9 | 1 | 1,27 | 1,55 | | 8:23822-58699 | 34878 | Degenerate | 38.0 | 38 |
| 84 |  |  |  | 2 | 0,28 |  |  | 13:1-7877 | 7877 | Degenerate | 35.5 | 14 |
| 85 | *D. hydrothermalis* DSM14728 | NC_020055.1 | 45.1 | 1 | 0,71 | 1,38 | | 220039-246518 | 26480 | Degenerate | 46.0 | 38 |
| 86 |  |  |  | 2 | 0,44 |  |  | 264063-280712 | 16650 | Degenerate | 41.0 | 25 |
| 87 |  |  |  | 3 | 0,23 |  |  | 7509-16126 | 8618 | Degenerate | 38.8 | 8 |
| 88 | *D. magneticus* RS-1 | NC_012796.1 | 62.8 | 1 | 0,54 | 1,31 | | 2787492-2816248 | 28757 | Degenerate | 64.1 | 34 |
| 89 |  |  |  | 2 | 0,14 |  |  | 1598917-1606756 | 7840 | Degenerate | 60.8 | 10 |
| 90 |  |  |  | 3 | 0,25 |  |  | 1651912-1665043 | 13132 | Degenerate | 44.3 | 8 |
| 91 |  |  |  | 4 | 0,19 |  |  | 3812347-3822640 | 10294 | Degenerate | 69.0 | 9 |
| 92 |  |  |  | 5 | 0,19 |  |  | 4745792- 4755972 | 10181 | Degenerate | 49.3 | 8 |
| 93 | *D.* *oxyclinae* DSM11498 | NZ_AQXE00000000.1 | 59.1 | 1 | 1,29 | 1,29 | | 1:255253-298505 | 43253 | Complete | 55.0 | 44 |
| 94 | *D. cuneatus* DSM11391 | NZ_AUCY00000000.1 | 53.5 | 1 | 0,51 | 1,21 | | 6:21787-39205 | 17419 | Degenerate | 54.6 | 24 |
| 95 |  |  |  | 2 | 0,70 |  |  | 9:76936-100771 | 23836 | Degenerate | 54.8 | 28 |
| 96 | *D. desulfuricans* [DSM 642](https://www.ncbi.nlm.nih.gov/genome/15717?genome_assembly_id=301186) | NZ_ATUZ00000000.1 | 57.4 | 1 | 0,66 | 1,10 | | 11:301623-324411 | 22789 | Degenerate | 59.8 | 29 |
| 97 |  |  |  | 2 | 0,44 |  |  | 11:716541-731554 | 15014 | Degenerate | 51.2 | 10 |
| 98 | *D. desulfuricans* [ATCC 27774](https://www.ncbi.nlm.nih.gov/genome/15717?genome_assembly_id=301187) | NC_011883.1 | 58.1 | 1 | 1,10 | 1,10 | | 277508-309276 | 31769 | Complete | 55.7 | 42 |
| 99 | *D. salexigens* DSM2638 | NC_012881.1 | 47.1 | 1 | 1,07 | 1,07 | | 2315338-2361506 | 46169 | Complete | 45.5 | 53 |
| 100 | *D.* *zosterae* DSM11974 | NZ_AUDC00000000.1 | 41.8 | 1 | 0,34 | 0,99 | | 10:126911-140903 | 13993 | Degenerate | 41.9 | 14 |
| 101 |  |  |  | 2 | 0,65 |  |  | 11:407097-434019 | 26923 | Degenerate | 41.9 | 29 |
| 102 | *D. magneticus* [IFRC170](https://www.ncbi.nlm.nih.gov/genome/1358?genome_assembly_id=171113) | NZ_JAGC00000000.1 | 64.7 | 1 | 0,58 | 0,91 | | 5:10256-38573 | 28318 | Degenerate | 63.9 | 32 |
| 103 |  |  |  | 2 | 0,33 |  |  | 5:48224-64398 | 16175 | Degenerate | 66.4 | 20 |
| 104 | *D.* *aminophilus* DSM12254 | NZ_AUMA00000000.1 | 66.2 | 1 | 0,23 | 0,80 | | 4:20345-28622 | 8278 | Degenerate | 68.3 | 8 |
| 105 |  |  |  | 2 | 0,57 |  |  | 19:13012-32703 | 19692 | Degenerate | 68.5 | 30 |
| 106 | *D. africanus* PCS | NZ_AOSV00000000.1 | 61.2 | 1 | 0,79 | 0,79 | | 63:67267-98453 | 31187 | Complete | 60.2 | 40 |
| 107 | *D. termitidis* HI1 | NZ_AZAO00000000.1 | 66.1 | 1 | 0,17 | 0,69 | | 2:6809-14189 | 7381 | Degenerate | 66.7 | 10 |
| 108 |  |  |  | 2 | 0,19 |  |  | 6:863784-872136 | 8353 | Degenerate | 67.3 | 15 |
| 109 |  |  |  | 3 | 0,14 |  |  | 7:67044-73337 | 6294 | Degenerate | 64.7 | 7 |
| 110 |  |  |  | 4 | 0,19 |  |  | 9:222471-230896 | 8426 | Degenerate | 65.3 | 8 |
| 111 | *D. brasiliensis* JCM12178 | NZ_BBCB00000000.1 | 59.7 | 1 | 0,61 | 0,61 | | 28:4203-26287 | 22085 | Degenerate | 61.1 | 24 |
| 112 | *D. frigidus* DSM17176 | NZ_JONL00000000.1 | 42.8 | 1 | 0,54 | 0,54 | | 12:1639-24526 | 22888 | Degenerate | 45.6 | 26 |
| 113 | *D. putealis* DSM16056 | NZ_AUBQ00000000.1 | 62.8 | 1 | 0,49 | 0,49 | | [4:295731-319243](http://phaster.ca/submissions/ZZ_9d5b8b4c7b#region_dna0) | 23513 | Degenerate | 65.8 | 34 |
| 114 | *D. dechloracetivorans* BerOc1 | NZ_LKAQ00000000.1 | 63.8 | 1 | 0,48 | 0,48 | | 04:2266245-2285871 | 19627 | Degenerate | 65.2 | 26 |
| 115 | *D. inopinatus* DSM10711 | NZ_AUBP00000000.1 | 49.2 | 1 | 0,28 | 0,45 | | 12:77918-94286 | 16369 | Degenerate | 49.9 | 18 |
| 116 |  |  |  | 2 | 0,17 |  |  | 17:89525-99426 | 9902 | Degenerate | 51.0 | 16 |
| 117 | *D. desulfuricans* ND132 | NC_016803.1 | 65.2 | 1 | 0,16 | 0,43 | | 1652158-1657915 | 5758 | Degenerate | 53.3 | 6 |
| 118 |  |  |  | 2 | 0,12 |  |  | 2638934-2643452 | 4519 | Degenerate | 66.1 | 6 |
| 119 |  |  |  | 3 | 0,15 |  |  | 2438616-2443963 | 5348 | Degenerate | 55.9 | 6 |
| 120 | *D. longus* DSM6739 | NZ_ATVA00000000.1 | 63.7 | 1 | 0,39 | 0,39 | | 13:177164-191691 | 14528 | Degenerate | 63.4 | 17 |
| 121 | *D. ferrireducens* DSM16995 | FNGA00000000.1 | 42.8 | 1 | 0,19 | 0,37 | | 1:98115-105581 | 7467 | Degenerate | 46.3 | 8 |
| 122 |  |  |  | 2 | 0,18 |  |  | 1:680280-687550 | 7271 | Degenerate | 42.6 | 8 |
| 123 | *D. magneticus* MBC34 | ALAO00000000.1 | 65.7 | 1 | 0,17 | 0,35 | | 303.133:16744-24517 | 7774 | Degenerate | 64.3 | 10 |
| 124 |  |  |  | 2 | 0,18 |  |  | 435.212:995-8978 | 7984 | Degenerate | 63.1 | 7 |
| 125 | *D. alcoholivorans* DSM5433 | NZ_JNJA00000000.1 | 64.7 | 1 | 0,13 | 0,33 | | 3:79563-86728 | 7166 | Degenerate | 63.3 | 9 |
| 126 |  |  |  | 2 | 0,20 |  |  | 7:63594-74297 | 10704 | Degenerate | 48.2 | 14 |
| 127 | *D.* *piezophilus* C1TLV30 | NC_020409.1 | 50.0 | 1 | 0,31 | 0,31 | | 1174880-1186253 | 11374 | Degenerate | 54.5 | 13 |
| 128 | *D. legallii* KHC7 | FNBX00000000.1 | 64.8 | 1 | 0,29 | 0,29 | | 18: 46636-54571 | 7936 | Degenerate | 66.4 | 10 |

**Supplementary Table S2** Complete prophages in *Desulfovibrio*

| **N°** | **Strain** | **Origin^a^** | **Prophage** | **Size (pb)** | **Family^b^** | **Proteins^c^** |
| --- | --- | --- | --- | --- | --- | --- |
| 1 | *D. aespoeensis* Aspo-2 | Env | 2 | 36770 | *Myo* | Lys hyp (WP_083808685.1) |
| 2 |  |  | 3 | 42929 | *Myo* | Lys hyp (WP_013514570.1) |
| 3 |  |  | 4 | 38064 | *Myo* | Lys (WP_013515329.1) |
| 4 | [*D. africanus* Walvis Bay](https://www.ncbi.nlm.nih.gov/genome/3152?genome_assembly_id=172937) | Env | 1 | 35440 | *Myo* | Hol hyp (WP_014258869.1) e Lys hyp (WP_014258893.1) |
| 5 | *D. africanus* [DSM2603](https://www.ncbi.nlm.nih.gov/genome/3152?genome_assembly_id=172938) | Env | 1 | 32937 | *Myo* | - |
| 6 | *D. africanus* PCS | Env | 1 | 31187 | *Sip* | - |
| 7 | *D.* *alaskensis* G20 | Env | 1 | 41050 | *Myo* | Lys hyp (WP_011366992.1) |
| 8 |  |  | 3 | 40230 | *Myo* | - |
| 9 |  |  | 4 | 48689 | *Myo* | Lys (WP_011367798.1) |
| 10 | *D. alaskensis* DSM16109 | Env | 1 | 40573 | *Myo* | - |
| 11 |  |  | 2 | 24453 | *Myo* | - |
| 12 | *D. alkalitolerans* DSM16529 | Env | 2 | 43877 | *Myo* | - |
| 13 | *D. bizertensis* DSM 18034 | Env | 3 | 36089 | *Myo* | Lys hyp (SKA81922.1) |
| 14 | *D. desulfuricans* [ATCC 27774](https://www.ncbi.nlm.nih.gov/genome/15717?genome_assembly_id=301187) | Env | 1 | 31769 | *Myo* | - |
| 15 | *D. desulfuricans* DSM7057 | Env | 1 | 43719 | *Pod* | Hol (SFW23071.1) e Lys (SFW23095.1) |
| 16 |  |  | 2 | 28623 | *Myo* | Lys (SFW30341.1) |
| 17 | [*D. desulfuricans* DSM17919](https://www.ncbi.nlm.nih.gov/genome/15717?genome_assembly_id=295293) | Env | 1 | 31194 | *Myo* | Hol hyp (SHI60426.1) |
| 18 | *D. fairfieldensis* CCUG45958 | Hum | 1 | 57140 | *Sip* | Hol hyp (WP_083521954.1) e Lys hyp (WP_062251836.1) |
| 19 |  |  | 2 | 38473 | *Myo* | Lys hyp (WP_062252149.1) |
| 20 |  |  | 3 | 30416 | *Myo* | Hol hyp (WP_062252788.1) e Lys hyp (WP_062252832.1) |
| 21 | *D. fructosivorans* JJ | Env | 1 | 37113 | *Sip* | Hol hyp (EFL53014.1) e Lys (EFL53013.1) |
| 22 |  |  | 2 | 50676 | *Sip* | Hol hyp (EFL53098.1) e Lys (EFL53099.1) |
| 23 | *D. gigas* ATCC19364 | Env | 1 | 44956 | *Sip* | Lys hyp (WP_021759525.1) |
| 24 |  |  | 2 | 33156 | *Sip* | Hol hyp (WP_021761111.1) |
| 25 |  |  | 3 | 56190 | *Sip* | Lys hyp (WP_051286217.1) |
| 26 | *D. indicus* J2 | Env | 1 | 36866 | *Myo* | Hol hyp (WP_066798947.1) e Lys (WP_066798949.1) |
| 27 |  |  | 2 | 32793 | *Myo* | - |
| 28 | *D.* *oxyclinae* DSM11498 | Env | 1 | 43253 | *Pod* | Lys hyp (292456-292824 complementar) |
| 29 | *D. piger*  FI11049 | Hum | 1 | 39418 | *Myo* | Hol hyp (WP_072331516.1) e Lys (WP_083575220.1) |
| 30 |  |  | 2 | 42600 | *Myo* | - |
| 31 |  |  | 5 | 40876 | *Myo* | Hol hyp (WP_072332501.1) e Lys (WP_083575254.1) |
| 32 | *D. piger* ATCC29098 | Hum | 2 | 37864 | *Myo* | - |
| 33 |  |  | 4 | 35309 | *Myo* | - |
| 34 | *D. piger* 64-16 | Hum | 1 | 47319 | *Myo* | Hol hyp (OLA87393.1) e Lys (OLA87415.1) |
| 35 | *D. salexigens* DSM2638 | Env | 1 | 46169 | *Myo* | Hol hyp (WP_081434591.1) |
| 36 | *D. vulgaris* Miyazaki | Env | 1 | 36988 | *Myo* | Lys (WP_012611872.1) e Lys hyp (WP_012611897.1) |
| 37 |  |  | 2 | 39403 | *Myo* | Hol (WP_012612411.1) e Lys (WP_012612412.1) |
| 38 | *D. vulgaris* Hildenborough | Env | 1 | 36674 | *Myo* | Hol (YP_009427.1) e Lys hyp (YP_009428.1) |
| 39 |  |  | 2 | 35621 | *Myo* | Hol hyp (YP_010350.1) e Lys (YP_010349.1) |
| 40 |  |  | 3 | 39411 | *Sip* | Hol (YP_010724.1) |
| 41 |  |  | 4 | 36552 | *Pod* | Hol hyp (YP_010945.1) |
| 42 |  |  | 5 | 35035 | *Myo* | Lys hyp (YP_011939.1) |
| 43 |  |  | 6 | 40679 | *Myo* | Hol (YP_012079.1) e Lys hyp (YP_012078.1) |
| 44 |  |  | 7 | 44367 | *Sip* | Hol (YP_011379.1) e Lys (YP_011378.1) |
| 45 | *D. vulgaris* DP4 | Env | 1 | 42742 | *Sip* | Hol (WP_081428991.1) e Lys (WP_011792031.1) |
| 46 |  |  | 2 | 36411 | *Myo* | Hol hyp (WP_011792275.1) |
| 47 |  |  | 4 | 34215 | *Myo* | Hol hyp (WP_011793063.1) |
| 48 | *D. vulgaris* RCH1 | Env | 1 | 40282 | *Myo* | Lys (WP_010938427.1) |
| 49 |  |  | 2 | 37208 | *Pod* | Hol hyp (WP_010939015.1) |
| 50 |  |  | 3 | 35035 | *Myo* | Lys (WP_010939994.1) |
| 51 |  |  | 4 | 40679 | *Myo* | Hol (WP_010940129.1) e Lys hyp (WP_010940128.1) |
| 52 |  |  | 5 | 34958 | *Sip* | Hol hyp (WP_010938799.1) |
| 53 |  |  | 6 | 42866 | *Sip* | Hol (WP_010939443.1) e Lys (WP_010939442.1) |

^a^ Strains were characterized in environmental (Env) and human (Hum).

^b^ Phages families were characterized in *Myoviridade* (*Myo*), *Siphoviridae* (*Sip*) and *Podoviridae* (*Pod*).

^c^ Protreins were characterized in lysozyme (Lys), holina (Hol), hypothetical (hyp).


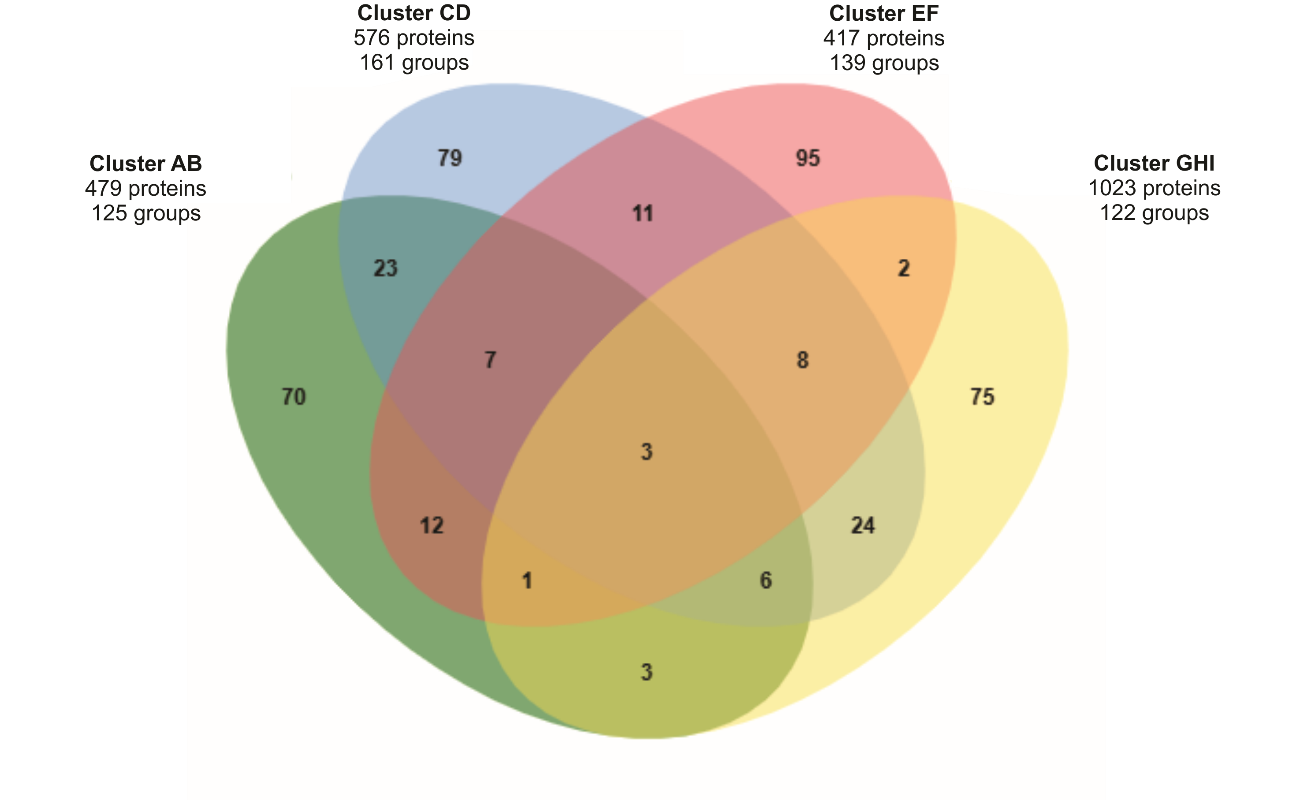


**Supplementary Fig. S1.** Sharing of proteins between *Desulfovibrio* complete prophages. The Venn Diagram was drafted with the total proteins. The numbers indicate protein groups shared between the four phylogenetic clusters (AB, CD, EF and GHI) of 53 *Desulfovibrio* prophages.


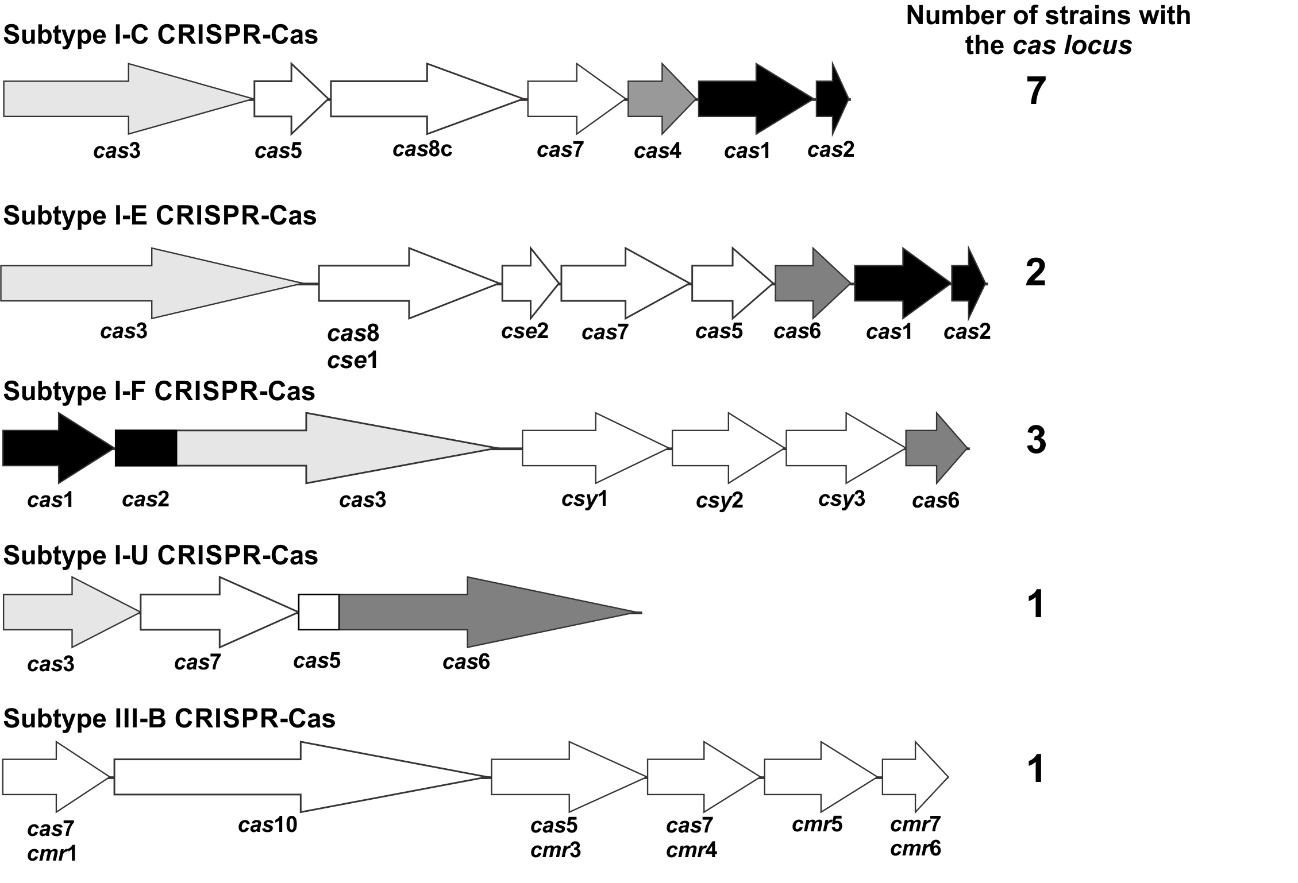


**Supplementary Fig. S2.** Architecture of the gene region of the CRISPR-Cas system in closed *Desulfovibrio* genomes. Each *cas* locus is represented according to a *Desulfovibrio* strain: *D. desulfuricans* ND132 (I-C), *D. alaskensis* G20 (I-E), *D. gigas* (I-F and I-U) and *D. hydrothermalis* (III-B). On the right is the number of genomes that have the cas locus type highlighted. The genes are colored according to their function: adaptation (black), pre-crRNA processing (dark gray), interference modulus (white) and target cleavage (light gray).

**Supplementary Table S3** CRISPR-Cas system in closed genomes of *Desulfovibrio* by CRISPRone

| **N°** | **Strain** | **CRISPR arrays (n° of spacers)** | **CRISPR-Cas systems type** | **Putative activity** |
| --- | --- | --- | --- | --- |
| 1 | *D. aespoeensis* Aspo-2 | 0 | I-incomplete | Inactive |
| 2 | *D. africanus* Walvis Bay | ND | ND | Inactive |
| 3 | *D. alaskensis* G20 | 1(20) | I-E | Active |
| 4 | *D. desulfuricans* ND132 | 1(100) | I-C | Active |
| 5 | *D. desulfuricans* ATCC 27774 | 1 (30) | I-E | Active |
| 6 | *D. fairfieldensis* CCUG45958 | ND | ND | Inactive |
| 7 | *D. gigas* ATCC19364 | 1(6) | I-F e I-U incomplete | Active |
| 8 | *D. hydrothermalis* DSM14728 | 6 (45) | III-B e I-F | Active |
| 9 | *D. indicus* J2 | 2(29) | I-C | Active |
| 10 | *D. magneticus* RS-1 | 2(52) | I-C e I-F | Active |
| 11 | *D. piezophilus* C1TLV30 | 0 | ND | Inactive |
| 12 | *D. piger* FI11049 | 1(3) | ND | Inactive |
| 13 | *D. salexigens* DSM2638 | 0 | I-incomplete | Inactive |
| 14 | *D. vulgaris* DP4 | 1(46) | I-C | Active |
| 15 | *D. vulgaris* Hildenborough | 1(28) | I-C | Active |
| 16 | *D. vulgaris* Miyazaki | 2(60) | I-C | Active |
| 17 | *D. vulgaris* RCH1 | 1(28) | I-C | Active |

ND: Not detected.
